# Diagnostic Algorithm for Metastatic Lymph Nodes of Differentiated Thyroid Carcinoma

**DOI:** 10.3390/cancers13061338

**Published:** 2021-03-16

**Authors:** Sae Rom Chung, Jung Hwan Baek, Young Jun Choi, Tae-Yon Sung, Dong Eun Song, Tae Yong Kim, Jeong Hyun Lee

**Affiliations:** 1 Department of Radiology and Research Institute of Radiology, University of Ulsan College of Medicine, Asan Medical Center, Seoul 05505, Korea; jserom@naver.com (S.R.C.); jehee23@gmail.com (Y.J.C.); jeonghlee@amc.seoul.kr (J.H.L.); 2 Department of Surgery, University of Ulsan College of Medicine, Asan Medical Center, Seoul 05505, Korea; tysung@amc.seoul.kr; 3 Department of Pathology, University of Ulsan College of Medicine, Asan Medical Center, Seoul 05505, Korea; hipuha@hanmail.net; 4 Department of Endocrinology and Metabolism, University of Ulsan College of Medicine, Asan Medical Center, Seoul 05505, Korea; tykim@amc.seoul.kr

**Keywords:** papillary thyroid carcinoma, neoplasm metastasis, ultrasonography, biopsy, fine-needle, thyroglobulin

## Abstract

**Simple Summary:**

Fine-needle aspiration cytology (FNAC) with measurement of thyroglobulin concentrations obtained through aspiration (FNA-Tg) is routinely used for the diagnosis of metastatic lymph nodes (LNs) from differentiated thyroid carcinomas. However, some areas of uncertainty remain, including the optimal FNA-Tg cutoff and its interpretation based on ultrasound (US) features. In this study, we evaluated the appropriate strategies for interpreting FNAC and FNA-Tg results based on the sonographic features of LNs. We confirmed that the malignancy rate of LNs found to be malignant by FNAC or elevated FNA-Tg was sufficiently high to be diagnosed as metastasis, regardless of the sonographic features. The malignancy rate of LNs with indeterminate or benign FNAC findings and low FNA-Tg were stratified according to their sonographic features. We propose a diagnostic algorithm, based on combined FNAC, FNA-Tg, and US features of LNs, for diagnosing metastatic LNs of differentiated thyroid carcinomas.

**Abstract:**

We aimed to evaluate appropriate strategies for interpreting fine-needle aspiration cytology (FNAC) and thyroglobulin concentrations obtained through aspiration (FNA-Tg) results based on the sonographic features of lymph nodes (LNs). Consecutive patients who underwent ultrasound-guided FNAC and FNA-Tg for metastatic LNs from differentiated thyroid carcinomas (DTCs) from January 2014 to December 2018 were reviewed retrospectively. LNs were categorized sonographically as suspicious, indeterminate, or benign. The optimal FNA-Tg cutoff for metastatic LNs was evaluated preoperatively, after lobectomy, and after total thyroidectomy. The diagnostic performances of FNA-Tg, FNAC, and their combination were analyzed based on the sonographic features of LNs. The malignancy rates of LNs were analyzed based on the sonographic features, FNAC, and FNA-Tg results. Of the 1543 LNs analyzed, 528 were benign, whereas 1015 were malignant. FNA-Tg increased the sensitivity and accuracy of FNAC for LNs. The malignancy rate of LNs found to be malignant by FNAC or elevated FNA-Tg ranged from 82% to 100%, regardless of the sonographic features. The malignancy rate of LNs with indeterminate or benign FNAC findings and low FNA-Tg were stratified according to their sonographic features. We propose a diagnostic algorithm, based on combined FNAC, FNA-Tg, and ultrasound features of LNs, for diagnosing metastatic LNs of DTCs.

## 1. Introduction

Patients with differentiated thyroid carcinoma (DTC) have an excellent prognosis. However, despite their relatively indolent clinical and biological behaviors, DTCs are frequently associated with cervical lymph node (LN) metastases at the time of diagnosis or during the postoperative follow-up period [1]. Accurate diagnosis of LN metastasis is important for patients with DTCs, with likely LN involvement taken into consideration when deciding whether to perform neck dissection or not and when predicting the patient prognosis. Particularly, lateral LN metastasis increases the risk of locoregional recurrence and decreases the rate of tumor-free survival among patients with PTC [2]. Thus, detection of lateral LN metastases during the initial operation is important for reducing reoperation rates and complications of reoperation [3].

Ultrasound (US)-guided fine aspiration cytology (FNAC) is the most useful technique for diagnosing nodal metastases, although inadequate cellularity or nonrepresentative sampling precludes diagnosis in up to 20% of specimens, including small metastatic lesions or those with partial involvement or cystic changes [4,5,6]. The diagnostic yield of FNA could improve by directly measuring the thyroglobulin (Tg) concentration in the washout fluid of the fine-needle aspirate (FNA-Tg) [7]. Current guidelines recommend that patients with DTC undergo biopsy of sonographically suspicious LNs to obtain cytology results and determine the FNA-Tg concentration [8,9,10].

Although FNA-Tg is important in assessing lesions suspected of being recurrent or metastatic, some areas of uncertainty remain, including the optimal FNA-Tg cutoff and its interpretation based on US features. Therefore, the aim of the present study was to determine the optimal FNA-Tg cutoff detecting malignant LNs based on the patient’s surgical status, compare the diagnostic performance of this cutoff with that of FNAC based on the US features of LNs, and propose a diagnostic algorithm to detect metastatic LNs of DTCs using combined FNAC, FNA-Tg, and US features of LNs.

## 2. Results

### 2.1. Study Population

A total of 1543 LNs in 1173 patients were included in our study. Of these 1543 LNs, 528 (34.2%) were benign and 1015 (65.8%) were metastatic, including 997 from classic-type papillary thyroid carcinoma, 15 from follicular variant papillary thyroid carcinoma, and 3 from follicular carcinoma. Of the 1543 LNs, 865 (56.0%) were obtained preoperatively, 63 (4.1%) were obtained after hemithyroidectomy, and 615 (39.9%) were obtained after total thyroidectomy. LN characteristics according to the final diagnosis are shown in Table 1. The mean FNA-Tg level was 45,266.8 ± 191,378.6 ng/mL for metastatic LNs and 21.3 ± 465.7 ng/mL for benign LNs. The mean serum Tg concentration was also significantly higher in patients with metastatic LNs than in those with benign LNs.

### 2.2. Optimal FNA-Tg Cutoff Values

**Figure 1:**
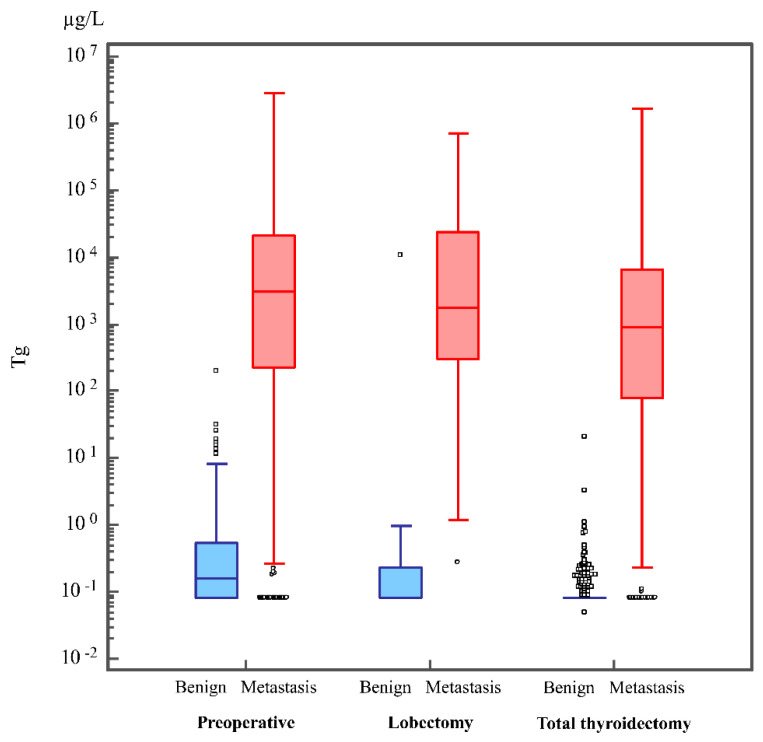
The distribution of FNA-Tg in benign and metastatic LNs obtained preoperatively, after lobectomy, and after total thyroidectomy. Boxplots represent median (line within box), 25th percentile (lower hinge) and its lower adjacent value (lower adjacent line), 75th percentile (upper hinge) and its upper adjacent value (upper adjacent line), and outside values (dots). FNA, fine-needle aspiration; LN, lymph node; Tg, thyroglobulin.

Boxplots showing the distribution of FNA-Tg levels in the benign and metastatic LNs obtained preoperatively, after lobectomy, and after total thyroidectomy are shown in Figure 1. The median FNA-Tg levels for metastatic and benign LNs were 3005 and 0.16 ng/mL, 1760 and 0.08 ng/mL, and 890 and 0.08 ng/mL in patients preoperatively, after lobectomy, and after total thyroidectomy, respectively.

**Figure 2:**
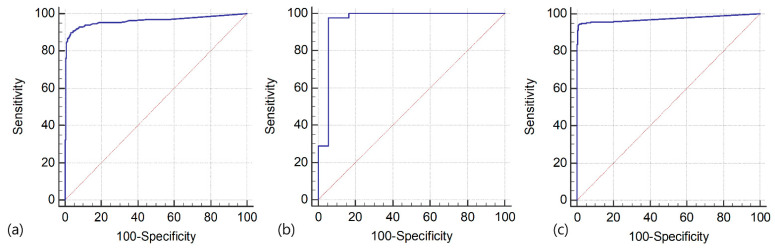
Receiver operating characteristic curves for FNA-Tg measurements in patients assessed preoperatively (**a**), after lobectomy (**b**), and after total thyroidectomy (**c**). The areas under the curves were 0.962, 0.958, and 0.974, respectively and *p* < 0.001; FNA, fine-needle aspiration; Tg, thyroglobulin.

The optimal FNA-Tg cutoff for detecting metastatic LNs were determined using the receiver operating characteristic (ROC) analysis. The optimal cutoff for LNs obtained preoperatively, after lobectomy, and after total thyroidectomy FNA-Tg were 8.3, 0.97, and 1.1 ng/mL, respectively, with area under the curve of 0.962, 0.958, and 0.974, respectively (Figure 2).

### 2.3. Comparison of the Diagnostic Performance of FNAC, FNA-Tg, and Combined FNAC and FNA-Tg

Table 2 compares the diagnostic performances of FNAC and combined FNAC and FNA-Tg according to the sonographic features of LNs. Only 2 of the 80 sonographically benign LNs were diagnosed as metastatic.

The positive predictive values of FNAC and FNA-Tg were 99.8–100% and 97.5–100%, respectively. Combined FNA-Tg to FNAC increases sensitivity of FNAC in the diagnosis of metastatic LNs, regardless of US features of LNs (*p* ≤ 0.004). Their specificities did not differ significantly (*p* ≥ 0.059). The negative predictive value of FNAC for LNs with cystic change, suspicious LNs without cystic change, and indeterminate LNs were 1.5%, 47.2%, and 86.7%, respectively. The combination of FNA-Tg and FNAC improved these values to 50%, 74.0%, and 95.6%, respectively.

Only one lesion was a false-positive on FNAC, with surgery confirming that this lesion was a suture granuloma with a foreign body reaction. There are nine LNs with false-positive results on FNA-Tg. Eight of nine LNs were obtained at the preoperative evaluation, and the false-positive result of FNA-Tg may be associated with elevated serum Tg levels in some preoperative patients with thyroid cancer.

There was only a single false-negative case on FNAC and FNA-Tg for LNs with cystic change, which was likely caused by mistargeting of the LN during FNA. For suspicious LNs without cystic change and indeterminate LNs, FNAC showed false-negative results of 52.8% and 13.3%, respectively. Combination of FNA-Tg and FNAC reduced these false-negative rates to 26% and 4.44%, respectively.

### 2.4. Malignancy Rates of LNs Based on Sonographic Features, FNAC, and FNA-Tg

Table 3 shows the malignancy rate of FNAC according to sonographic features, as well as the FNA-Tg results of the LNs. Of the 805 LNs diagnosed as malignant by FNAC, 804 (99.9%) were metastatic LNs with the malignancy rate ranging from 97.1% to 100%. The malignancy rates of LNs with elevated FNA-Tg were higher than 87.0%, regardless of FNAC or sonographic feature of LNs. The malignancy rate of LNs with cytologically indeterminate results and low FNA-Tg was 8.9–46.7%. The malignancy rates of LNs with cytologically benign results and low FNA-Tg with sonographically suspicious and indeterminate were 22.9% and 3.8%, respectively.

## 3. Discussion

This study showed that the optimal cutoffs for LNs obtained preoperatively, after lobectomy, and after total thyroidectomy FNA-Tg were 8.3, 0.97, and 1.1 µg/L. Adding FNA-Tg to FNAC improved the sensitivity and accuracy for the diagnosis of LNs that were sonographically indeterminate and suspicious. The analysis of malignancy rates of LNs based on their sonographic features, FNAC results, and FNA-Tg cutoff resulted in stratification of the risk of malignancy. Based on these results, we propose an algorithm, based on a combination of FNAC, FNA-Tg, and US features of LNs, for the diagnosis of metastatic LNs of DTCs.

Tg is a glycoprotein produced specifically by the follicular cells of the thyroid, regardless of whether the cells are benign or malignant [11]. This association of Tg concentration with thyroid follicular cells allows for the postoperative monitoring and diagnosis of metastatic LNs in patients with DTCs. Although FNA-Tg has been found to improve the evaluation of suspicious LNs in DTC patients, the cutoff for FNA-Tg varies among studies [7,12,13,14,15,16,17,18,19,20,21]. Serum Tg is a potential source of bias because Tg in the peripheral blood can contaminate aspirated fluid during FNA [12,22]. Because serum Tg is associated with the presence of follicular cells and tumor burden, even when not indicative of pathological status [23,24], FNA-Tg cutoff should reflect the surgical status of patients and the presence of residual thyroid tissue. Although the FNA-Tg cutoffs have been determined for detecting metastatic LNs, previous studies have been limited by small numbers of included participants [12,17,18,22,25] or because they did not consider the patients’ surgical status [13,19]. Thus, the present study determined separate FNA-Tg cutoff preoperatively, after lobectomy, and after total thyroidectomy in a large study population. The optimal FNA-Tg cutoff was higher for patients evaluated preoperatively (8.3 µg/L) than after surgery, which was consistent with previous studies [16,21,22,25], but was similar after lobectomy (0.97 µg/L) and after total thyroidectomy (1.1 µg/L).

The cystic appearance of LNs is a characteristic finding of metastatic DTC. Cystic metastatic LNs have shown high false-negative rates on FNAC, which can be reduced by the addition of FNA-Tg measurements [13,14,26]. However, the ability of FNA-Tg for the diagnosis of sonographically suspicious LNs without cystic changes or indeterminate LNs has not been investigated. Metastatic LNs with suspicious features on US are distinguished by gross tumor cell implantation, whereas metastatic LNs with indeterminate sonographic features are likely to contain micrometastases that are not large enough to produce any characteristic changes on US [8]. Consequently, the diagnostic performances of FNAC and FNA-Tg may differ according to the sonographic appearance of LNs. In our study, we found that adding FNA-Tg increased the sensitivity and accuracy of FNAC in the diagnosis of sonographically suspicious and indeterminate LNs. These results are consistent with findings showing that FNA-Tg increased the accuracy of the diagnosis of LNs without suspicious features [14]. By contrast, another study reported that FNA-Tg did not enhance the ability of diagnosing LNs without suspicious features, suggesting that FNA-Tg is not useful for diagnosing LNs without suspicious features [13]. This discrepancy may be a result of the application of the same cutoff for pre- and postoperative patients in that study.

**Figure 3:**
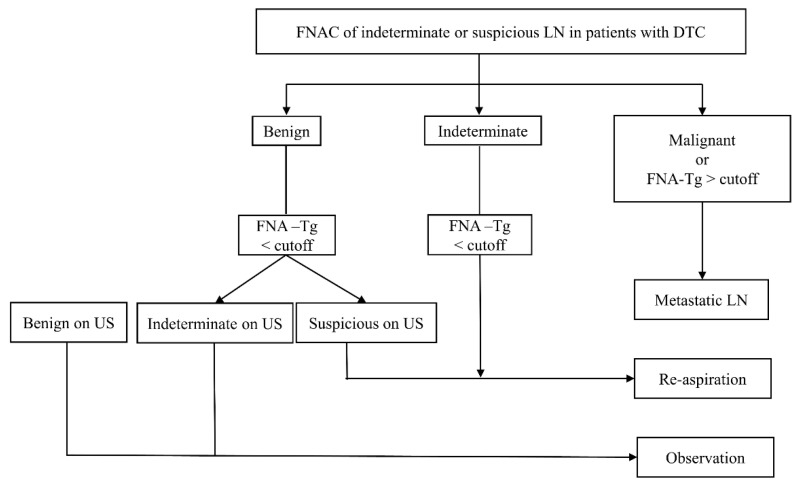
Diagram showing the algorithm for the diagnosis and management of metastatic LNs from DTC. DTC, differentiated thyroid carcinoma; FNA, fine-needle aspiration; LN, lymph node; Tg, thyroglobulin; US, ultrasound.

The malignancy rates of LNs were analyzed based on the sonographic features, FNAC results, and FNA-Tg cutoff, resulting in the development of the diagnostic algorithm shown in Figure 3. First, because sonographically benign LNs have a very low malignancy rate (2.5%), FNA is not recommended. This is consistent with several current guidelines [8,9,10]. Sonographically suspicious and indeterminate LNs with cytologically malignant or with elevated FNA-Tg can be diagnosed as metastatic LNs. The malignancy rates of LNs with cytologically indeterminate or benign results and low FNA-Tg were further stratified according to the sonographic features of LNs. Re-aspiration is recommended for LNs with cytologically indeterminate results and low FNA-Tg, because the malignancy rate ranges from 8.9% to 46.7%. LNs with cytologically benign results, low FNA-Tg, and sonographically indeterminate characteristics should undergo observation because of their low malignancy rates of 3.8%. However, if these LNs have suspicious sonographic findings, re-aspiration is recommended because their malignancy rate is 22.9%.

One of the limitations of the present study was its retrospective design. In addition, although our study included large numbers of patients and LNs, all patients were recruited at a single tertiary referral center. This could result in selection bias among the study population. It should also be noted that different devices for measuring FNA-Tg among institutions and tissue samples obtained by FNA can contain variable cell content and that diluting volumes of saline may not always precisely correspond to 1 mL. Therefore, our cutoff may not be applicable to patients from other institutions, and it may be difficult to compare results from different studies. Finally, there may have been interobserver variability in categorizing LNs as benign, indeterminate, and suspicious based on US. Prospective, multicenter studies would help to validate the generalizability of our findings.

## 4. Materials and Methods

### 4.1. Patient Selection

**Figure 4:**
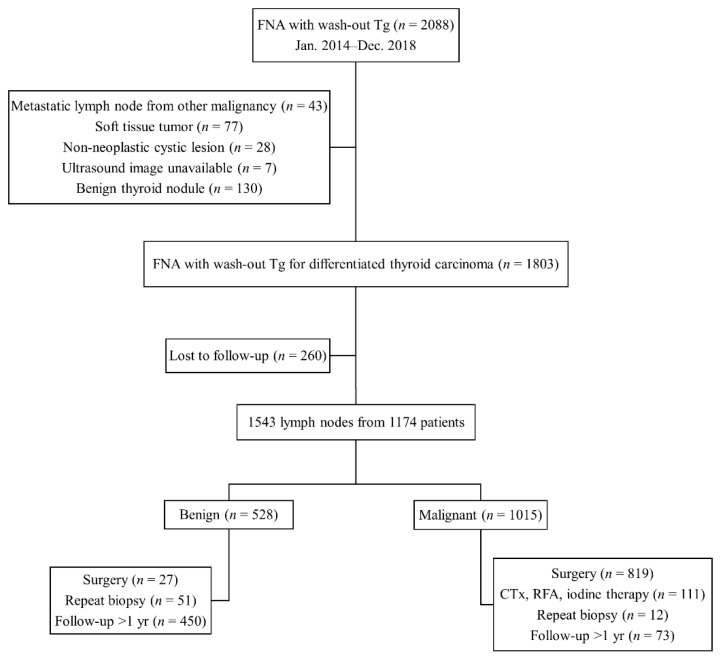
Flowchart showing the study population.

This retrospective study was approved by the Institutional Review Board of our institution approval (No. 2020-1508, 24 February 2020), and patient consent was waived due to retrospective study. Consecutive patients who had undergone US-guided FNA of LNs with measurement of FNA-Tg at our institution from January 2014 to December 2018 were included. Patients were included if they (i) were diagnosed with DTC and (ii) had metastatic LNs at final diagnosis. Patients were excluded if (i) they were not diagnosed with DTC, (ii) LNs were diagnosed as metastatic from other malignancies, (iii) evaluated lesions were not LNs, or (iv) they did not undergo subsequent surgical resection or follow-up imaging for at least 1 year. Finally, 1543 LNs from 1174 patients were included in the study population (Figure 4).

### 4.2. US and US-FNA

All patients underwent US examination of the neck using the HDI 5000 or IU22 scanner (Philips Medical Systems, Bothell, WA, USA) with a 12.5-MHz linear phased-array transducer. US-guided FNA of suspicious or indeterminate LNs, regardless of their size, was performed by radiologists. Sonographically benign LNs were defined by the presence of echogenic hilum or hilar vascularity in the absence of suspicious findings. Sonographically suspicious LNs were diagnosed if any of the following features was present: calcification, cystic change, hyperechogenicity, or peripheral or diffuse increased vascularity on color Doppler imaging. Sonographically indeterminate LNs did not have benign or suspicious LN imaging features (neither echogenic hilum nor hilar vascularity in the absence of suspicious findings), according to the Korean Society of Thyroid Radiology guidelines. Sonographically benign LNs were examined at the clinician’s request, mainly because of their enlarged size.

FNA was performed under US guidance with the free hand technique using a 23-gauge needle connected to a 10-mL syringe. FNA specimens were prepared from direct smears or liquid-based cytology. The specimen was smeared onto a slide and immediately fixed in 95% ethanol using the direct smear method. With the liquid-based cytology method, specimens were prepared using the ThinPrep 2000 Processor (Hologic Co., Marlborough, MA, USA). The same needle and syringe were rinsed with 1 mL normal saline, and Tg was measured in the washout fluid (FNA-Tg). If aspirates were serous fluid, Tg was measured in this fluid without adding saline [18,27]. Cytology findings were interpreted by cytopathologists specialized in thyroid cytology. The cytology results were grouped into three categories: malignant, benign, and indeterminate [15].

### 4.3. Measurement of Tg

Serum Tg and FNA-Tg were measured using the immunoradiometric method (Tg-PluS RIA kit; BRAHMS AG, Henningsdorf, Germany) with a functional sensitivity (coefficient of variation of 20%) of 0.2 µg/L and an analytical sensitivity of 0.08 µg/L.

### 4.4. Reference Standard

LNs were finally diagnosed as metastatic if they showed malignant FNA cytology satisfying at least one of the following criteria: (i) confirmation based on the surgical specimen, (ii) subsequent repeat FNA or core-needle biopsy, or (iii) follow-up with imaging after more than 1 year. LNs with benign or indeterminate FNA cytology and deemed free of metastatic disease by the same criteria were finally diagnosed as benign.

### 4.5. Statistical Analysis

Continuous variables were compared by Student’s *t*-tests, and categorical variables by the chi-square or Fisher’s exact test. Optimal FNA-Tg cutoff concentrations determining malignant LNs were evaluated preoperatively, after lobectomy, and after total thyroidectomy by ROC curve analysis and maximization of the Youden index (sensitivity + specificity − 1).

The diagnostic performances of FNAC, FNA-Tg, and their combination, including their sensitivity, specificity, and diagnostic accuracy, according to the sonographic characteristic of LNs, were compared by McNemar’s test. Associations were considered statistically significant at an α level of 0.025 (0.05/2); i.e., using a Bonferroni correction due to multiple comparisons issue. The malignancy rates of LNs were analyzed based on their sonographic features, FNAC results, and FNA-Tg cutoff.

All statistical analyses were performed using SAS, version 9.4 (SAS Institute, Cary, NC, USA) and MedCalc version 19.1 (MedCalc Software, Mariakerke, Belgium).

## 5. Conclusions

In conclusion, measuring FNA-Tg is useful for improving the detection of metastatic LNs from DTCs, regardless of their sonographic features. The evaluation of these metastatic LNs based on FNA-guided biopsy should include needle-wash Tg measurements. We propose a diagnostic algorithm for the diagnosis of metastatic LNs from DTCs. This algorithm, which includes FNAC, FNA-Tg, and US features of LNs, is applicable to most patients with this disease.

## Figures and Tables

**Table 1:** Demographic and clinical characteristics of patients and lymph nodes.

Characteristic	Benign	Metastatic	*p*-Value
No. of patients	418	755	
Age (years)	50.8 ± 13.2	49.4 ± 14.7	0.003
Sex Male Female	107 (25.6%) 311 (74.4%)	266 (35.2%) 489 (64.8%)	0.0004
No. of nodules	528	1015	
Preoperative evaluation Lobectomy Total thyroidectomy	261 (49.4%) 18 (3.4%) 249 (47.2%)	604 (59.5%) 45 (4.4%) 366 (36.1%)	0.0001
Sonographic diagnosis Benign Indeterminate Suspicious	78 (14.8%) 371 (70.2%) 79 (15.0%)	2 (0.2%) 143 (14.1%) 870 (85.7%)	<0.0001
Size Short axis diameter Longest diameter	0.5 ± 0.2 1.1 ± 0.5	0.6 ± 0.5 1.1 ± 0.7	<0.0001 0.242
FNA cytology Benign Malignant Indeterminate	472 (89.4%) 1 (0.2%) 55 (10.4%)	92 (9.1%) 804 (79.2%) 119 (11.7%)	<0.0001
FNA-Tg, ng/mL	21.3 ± 465.7	45,266.8 ± 191,378.6	<0.0001
Serum Tg, ng/mL	9.2 ± 34.8	37.9 ± 201.0	0.001

FNA, fine-needle aspiration; Tg, thyroglobulin.

**Table 2:** Diagnostic performances of fine-needle aspiration and thyroglobulin measurements for the diagnosis of lymph node metastases according to sonographic features.

Variables	Suspicious LN with Cystic Changes			Suspicious LN without Cystic Changes			Indeterminate Lymph Nodes		
	FNAC	FNA-Tg	Combined	FNAC	FNA-Tg	Combined	FNAC	FNA-Tg	Combined
Sensitivity	70.9 (161/227) [64.6–76.7]	99.6 * (226/227) [97.6–9.99]	99.6 ^†^ (226/227) [97.6–9.99)	86.6 (557/643) [83.8–89.2]	91.0 * (585/643) [88.5–93.1]	96.1 ^†^ (618/643) [94.3–97.5]	60.1 (86/143) [51.6–68.2]	81.8 * (117/143) [74.5–87.8]	88.1 ^†^ (126/143) [81.7–92.9]
Specificity	100 (1/1) [2.5–100]	100 (1/1) [2.5–100]	100 (1/1) [2.5–100]	98.7 (77/78) [93.1–100]	92.3 (72/78) [84.0–97.1]	91.0 (71/78) [82.4–96.3]	100 (371/371) [99.0–100]	99.2 (368/371) [97.7–99.8]	99.1 (368/371) [97.7–99.8]
PPV	100 (161/161) [100–100]	100 (226/226) [100–100]	100 (226/226) [100–100]	99.8 (557/558) [98.8–100]	99.0 (585/591) [97.8–99.5]	98.9 (618/625) [97.8–99.4]	100 (86/86) [100–100]	97.5 (117/120) [92.7–99.2]	97.7 (126/129) [93.1–99.2]
NPV	1.5 (1/67) [1.2–1.8]	50 (1/2) [12.4–87.6]	50 (1/2) [12.4–87.6]	47.2 (77/163) [42.3–52.2]	55.4 (72/130) [49.1–61.5]	74.0 (71/96) [65.8–80.8]	86.7 (371/428) [84.2–88.8]	93.4 (368/394) [90.9–95.3]	95.6 (368/385) [93.3–97.1]
Diagnostic accuracy	71.1 (162/228) [64.7–96.9]	99.6 * (227/228) [97.6–100]	99.6 ^†^ (227/228) [97.6–100]	87.9 (634/721) [85.3–90.2]	91.1 (657/721) [88.8–93.1]	95.6 ^†^ (689/721) [93.8–96.9]	88.9 (457/514) [85.9–91.5]	94.4 * (485/514) [92.0–96.2]	96.1 ^†^ (494/514) [94.1–97.6]
False-positive	0 (0/161)	0 (0/226)	0 (0/226)	0.2% (1/558)	1% (6/591)	1.1% (7/625)	0 (0/86)	2.5% (3/120)	2.3% (3/129)
False-negative	1.5% (1/67)	50% (1/2)	50% (1/2)	52.8% (86/163)	44.6% (58/130)	26% (25/96)	13.3% (57/428)	6.6% (26/394)	4.4% (17/385)

Data are percentage with proportion in parentheses and 95% confidence index in brackets unless otherwise indicated. Cutoff for Tg-FNA were 8.3 µg/L preoperatively, 0.97 µg/L following lobectomy, and 1.1 µg/L following total thyroidectomy. * *p* < 0.025 for comparisons of FNAC and FNA-Tg. ^†^ *p* < 0.025 for comparisons of FNAC and combined FNAC and FNA-Tg. LN, lymph node; FNA, fine-needle aspiration; Tg, thyroglobulin; PPV, positive predictive value; NPV, negative predictive value.

**Table 3:** Malignancy rate of lymph nodes (LNs) according to the sonographic feature, fine-needle aspiration cytology results, and washout thyroglobulin levels.

FNA Result	Sonographic Feature of LN	Malignancy Rate		
		Total	FNA-Tg > Cutoff	FNA-Tg < Cutoff
Malignant	Suspicious Indeterminate	99.9% (718/719) 100% (86/86)	100% (685/685) 100% (77/77)	97.1% (33/34) 100% (9/9)
Benign	Suspicious Indeterminate	45.7% (59/129) 9.1% (33/363)	87.0% (40/46) 87.0% (20/23)	22.9% (19/83) 3.8% (13/340)
Indeterminate	Suspicious Indeterminate	92.1% (93/101) 36.9% (24/65)	100% (86/86) 100% (20/20)	46.7% (7/15) 8.9% (4/45)

Cutoff values for Tg-FNA were 8.3 µg/L preoperatively, 0.97 µg/L after lobectomy, and 1.1 µg/L after total thyroidectomy. FNA, fine-needle aspiration; LN, lymph node; Tg, thyroglobulin.

## Data Availability

Data sharing not applicable.
